# Functional and Rheological Properties of Gluten-Free Flour Blends from Brown *Eragrostis tef* (Zucc.) Trotter and *Glycine max* (L.) Merr

**DOI:** 10.3390/molecules30244817

**Published:** 2025-12-18

**Authors:** Shewangzaw Addisu Mekuria, Damian Marcinkowski, Joanna Harasym

**Affiliations:** 1 Department of Biotechnology and Food Analysis, Wroclaw University of Economics and Business, Komandorska 118/120, 53-345 Wroclaw, Poland; shewangzaw.mekuria@ue.wroc.pl; 2 College of Veterinary Medicine and Animal Sciences, University of Gondar, Gondar P.O. Box 196, Ethiopia; 3 Department of Inorganic Chemistry, Wroclaw University of Economics and Business, Komandorska 118/120, 53-345 Wroclaw, Poland; damian.marcinkowski@ue.wroc.pl; 4 Adaptive Food Systems Accelerator-Science Centre, Wroclaw University of Economics and Business, Komandorska 118/120, 53-345 Wroclaw, Poland

**Keywords:** teff, soybean, gluten-free flour, functional properties, pasting behavior, rheology, texture profile, composite flour

## Abstract

The increasing prevalence of celiac disease and demand for nutritious gluten-free alternatives have driven interest in cereal–legume composite flours. This study examined the functional, rheological, and textural properties of gluten-free flour blends formulated from brown (red) teff (*Eragrostis tef* (Zucc.) Trotter) and soybean (*Glycine max* (L.) Merr.) at different ratios (100:0, 90:10, 80:20, 70:30, 60:40, 0:100). Absorptive characteristics, particle size distribution, pasting behaviour, viscoelastic properties through oscillatory rheology, and texture profile analysis were evaluated. Soybean flour exhibited higher water holding capacity (5.54 g/g) and water solubility index (40.18%), while teff demonstrated notable water absorption index (5.62 g/g) and swelling power (6.18 g/g). Particle size analysis revealed that coarse fractions enhanced water binding and solubility, whereas fine fractions favoured hydration and swelling. Pasting properties showed that teff achieved a peak viscosity of 12,198 mPas in water, significantly reduced to 1839 mPas with AgNO_3_. Pure teff exhibited the highest storage modulus (1947.98 Pa) and hardness (7.60 N), whereas the incorporation of soybeans progressively softened the texture. The complementary functional properties of teff and soybean demonstrate promising potential for developing nutritionally enhanced, protein-enriched gluten-free products, with solvent selection and blending ratios serving as critical optimization parameters for specific food applications.

## 1. Introduction

The demand for gluten-free diets has increased significantly in recent years due to the rising prevalence of celiac disease, gluten sensitivity, and consumer demand for healthy dietary options [1]. Even minimal consumption of gluten-containing foods can cause serious health problems for people with celiac disease and other gluten-related conditions, including wheat allergy and non-celiac gluten sensitivity [2]. There is also an increased demand for nutraceuticals that support gluten-free diets as part of a balanced nutritional approach. However, formulating gluten-free products remains challenging due to gluten’s critical role in providing dough elasticity, gas retention, and favourable textural characteristics [3]. Therefore, an investigation into both functionally and nutritionally adequate food products developed from nutrient-rich cereals and legumes as alternative flours is essential [4].

Teff (*Eragrostis tef* (Zucc.) Trotter), originating from Ethiopia, has gained considerable attention for its naturally health-promoting characteristics and nutrient-packed gluten-free composition, making it a valuable stable food crop [5,6]. Its nutritional and nutraceutical importance, combined with its low glycemic index, make it suitable for blending with other food sources [6,7]. Due to its gluten-free nature and functional characteristics, individuals with celiac disease are increasingly preferring teff-based products, and it is being selected for gluten-free product development to enhance texture, moisture retention, and shelf life [8]. In comparison, soybeans (*Glycine max* (L.) Merr.) are a gluten-free legume with high protein quality and antioxidant potential, which can be blended with cereal-based foods to enhance their nutritional profile, texture, and antioxidant properties [9,10]. Therefore, combining teff and soybean offers a promising approach for developing gluten-free flours that enhance the nutritional, antioxidant, sensory, and consumer acceptability properties of food products [11,12].

However, in addition to nutritional, antioxidant, and functional properties, consideration must also be given to the granulometric distribution, pasting behaviour, viscoelastic performance, and texture profile of individual and blended flours. These characteristics are critical in determining the processability and consumer acceptability of composite flours. Particle size distribution affects hydration capacity, mixing behaviour, and dough uniformity [7]. Therefore, optimizing milling and blending procedures is essential for achieving uniformity in gluten-free flours.

Studies have shown that teff typically exhibits strong pasting characteristics due to higher starch content and hydrolysis capacity, while soybean proteins can modify starch gelatinization and viscoelasticity [13,14]. Blending legumes with cereals enhances phenolic content and antioxidant activity; however, higher substitution levels may affect gel strength and resilience [9,15]. The pasting characteristics (thickening capacity, gelation behaviour, and baking potential) of composite flours are determined by the properties of individual flour components. Due to its high starch content, teff exhibits higher peak and breakdown viscosity [13,16], whereas soybeans restrict starch swelling, reducing peak viscosity but improving thermal stability [14]. Viscoelastic properties are another important consideration in gluten-free systems, where legume proteins can reinforce network formation [3]. However, excessive substitution of starch with protein may reduce elasticity and weaken the gel structure [9]. Texture attributes are critical determinants of further consumer acceptability; blending cereal–legume flours can enhance hardness and chewiness [3], though the proportion of legume flour must be carefully optimized to avoid disrupting starch gel strength [16]. Therefore, balancing teff–soybean blends is expected to optimize textural performance by combining protein strength with starch flexibility. In-depth rheological analysis of novel blends can build knowledge for the precise design of specific functionalities (such as chewiness or crispiness) in more complex food matrices.

Despite expanding research on cereal–legume blends, no comprehensive study has investigated the rheological, textural, and particle size aspects of teff–soybean composite flours, particularly under various ionic systems. Therefore, this study examines the functional, pasting, rheological, and textural characteristics of starch–protein interactions of gluten-free flour blends formulated from (*Eragrostis tef* (Zucc.) Trotter) and soybean (*Glycine max* (L.) Merr.) using water and silver nitrate [17] as solvents.

## 2. Results and Discussion

### 2.1. Absorptive Characteristics

Significant differences (*p* < 0.05) were observed in functional properties across all cereal–legume blend formulations. The absorptive characteristics of teff, soybean, and their blends are shown in Table 1.

Pure soybean flour exhibited the highest WHC (5.54 g/g), while pure teff flour showed the lowest (4.60 g/g). The composite blends displayed intermediate WHC values ranging from 4.69 to 4.93 g/g, with most formulations showing no significant difference (*p* > 0.05) from each other, although 80T20S (4.93 g/g) was statistically grouped as ‘ab’, indicating slight differentiation. The higher WHC in soybean flour is attributed to its elevated protein and fibre content [18], which provides abundant hydrophilic groups capable of binding water, whereas the starch-rich teff composition offers fewer hydrophilic binding sites. Similar trends have been reported for cereal–legume blends [19,20].

Both teff (2.85 g/g) and soybean (2.63 g/g) exhibited relatively high WAC values when measured individually. However, blends exhibited lower WAC values (0.59 to 0.77 g/g), representing an approximately 75% reduction compared to pure flours. This substantial decrease suggests that blending disrupts the water absorption mechanisms present in individual flours. The present finding aligns with reports from [21,22], who observed similar WAC declines upon blending maize/wheat with legume flours. Similarly, OAC (g/g) showed no significant difference (*p* > 0.05) between pure soybean (1.89) and teff flour (1.84), both statistically grouped as ‘d’. However, composite flours exhibited significantly lower OAC values (*p* < 0.05), ranging from 0.02 to 0.28 g/g. This reduction could be attributed to reduced exposure of hydrophobic protein sites during the blending process, potentially due to protein–starch interactions that mask lipophilic binding sites [23,24].

The hydrophilic–lipophilic index (HLI) values revealed an interesting pattern. Specific blends, particularly 90T10S (32.07) and 60T40S (21.42), exhibited significantly higher (*p* < 0.05) HLI values compared to pure teff (1.56) and soybean (1.39). Since HLI is calculated as WAC/OAC, these elevated values primarily result from the extremely low OAC values in these blends (0.02 and 0.04 g/g, respectively), rather than from increased water absorption capacity. This could result from the formation of protein–starch complexes that preferentially bind water while excluding oil [25].

Teff flour demonstrated the highest WAI (5.62 g/g), while soybean exhibited the lowest (3.15 g/g). The composite blends showed intermediate WAI values ranging from 4.50 to 4.73 g/g, with no significant differences among the various blend ratios (all grouped as ‘b’). This plateau effect suggests that once teff and soybean are combined, the hydration behaviour stabilizes at an intermediate level regardless of the specific blend ratio within the tested range. The reduction from pure teff to blends indicates that soybean proteins interfere with starch gelatinisation to some degree, though this effect reaches equilibrium quickly even at low substitution levels. In contrast, the WSI exhibited a clear progressive trend inversely related to teff content. Soybean flour showed the highest WSI (40.18%), while pure teff displayed the lowest (9.05%). The blend formulations demonstrated progressively increasing WSI values (10.01–19.93%) with increasing soybean content, with each increment in soybean proportion resulting in higher solubility. This trend is attributed to the contribution of soluble soybean proteins and oligosaccharides [26,27,28], which demonstrated that protein–polysaccharide interactions increase the soluble fraction in aqueous systems.

Swelling power (SP) showed significant differences (*p* < 0.05) between pure and blended flours. Teff exhibited the highest SP (6.18 g/g), consistent with reports by Boka et al. [29] and reflecting its starch-rich composition and strong hydration capacity. Soybeans displayed a lower SP (5.27 g/g) due to their limited starch content. The composite blends displayed intermediate SP values between 5.26 and 5.61 g/g, with minimal variation among formulations, demonstrating that swelling behaviour is primarily governed by the starch content contributed by teff, with soybean having a moderating effect.

### 2.2. Correlation Between Particle Size and Functional Properties

Particle size distribution and the correlation of absorptive characteristics with particle size are shown in Table 2. Coarse particles (>0.355 mm), predominantly from soybean flour, showed strong positive correlations with WHC (r = 0.64) and WSI (r = 0.82), confirming that protein- and fibre-rich soybean particles enhance water binding and solubility. However, these coarse fractions exhibited a negative correlation with WAI (r = −0.76), indicating reduced efficiency in starch hydration. This pattern suggests that coarse particles, while rich in proteins and lipids that effectively bind water and release soluble components, contain less accessible starch for hydration and swelling [16,30,31].

Medium-sized particles (0.25–0.355 mm) demonstrated positive correlations with WAI (r = 0.78) and SP (r = 0.35), revealing that moderate granule sizes optimize starch–water interactions and enhance gelatinisation [32]. Conversely, this particle fraction showed strong negative association with WSI (r = −0.81), suggesting that medium-sized particles promote water binding within the starch matrix rather than releasing soluble components into the aqueous phase.

Fine particles (<0.25 mm) exhibited positive associations with HLI (r = 0.67) and WAI (r = 0.52), but negative correlation with WSI (r = −0.74). This indicates that fine teff-rich fractions, containing higher proportions of starch and dietary fibre, promote starch swelling and water absorption but contribute less to the solubilization of flour components [16,33]. The negative correlation with WSI suggests that fine particles bind water tightly within their structure rather than releasing soluble materials.

The results suggest that coarse soybean-rich fractions favour water holding capacity and solubility, whereas fine teff-rich fractions enhance hydration and swelling properties, with medium fractions providing balanced functionality. This pattern aligns with previous research [16,34], which reported that particle size modifies the exposure of starch and protein matrices, thereby altering water and oil binding interactions in cereal–legume blends.

### 2.3. Pasting Properties

**Figure 1:**
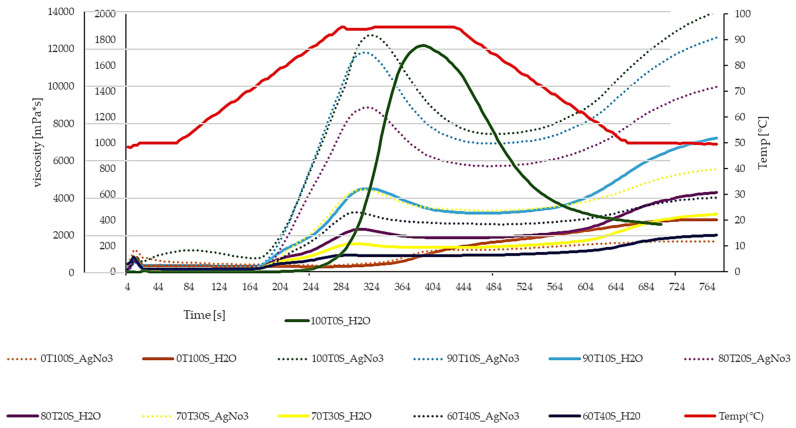
RVA analysis of the teff–soybean blend using solvent distilled water (H_2_O) and silver nitrate (AgNO_3_). First Y-axis on the left is for the 1000T0S_H_2_O sample, and the second Y-axis on the left is for the rest of the samples.

Table 3 and Figure 1 present the pasting properties of teff, soybean, and their blends using distilled water and silver nitrate (AgNO_3_) as solvents, revealing significant differences (*p* = 0.05) across all parameters. Silver nitrate is an amylase inhibitor to block endogenous alpha-amylase activity, allowing for the isolation of starch pasting behaviour (swelling, peak viscosity, breakdown) by preventing starch breakdown due to enzymatic hydrolysis [17]. Teff flour treated with distilled water exhibited the highest peak viscosity (PV) of 12,198 mPa·s, which was reduced to 1839 mPa·s when AgNO_3_ was used as the solvent.

The results revealed differences between the two solvent systems, particularly for formulations rich in teff. Pure teff flour (100T0S) exhibited notably higher peak viscosity in distilled water (12,198 ± 133 mPa·s) compared to AgNO_3_ solution (1839 ± 30 mPa·s). This pattern contrasts with observations reported by Rahman et al. (2020) in rice flour, where AgNO_3_ typically increased peak viscosity by inhibiting α-amylase-mediated starch degradation [35]. The inverse relationship observed in teff suggests either minimal endogenous α-amylase activity or alternative interactions between silver ions and teff starch components [36,37]. According to Alemneh et al. (2022), teff starch possesses unique structural characteristics that influence its swelling and gelatinization behaviour, which may explain this atypical response to AgNO_3_ [38].

The breakdown viscosity values are consistent with these solvent-dependent differences. In distilled water, teff flour demonstrated exceptionally high breakdown (9725 ± 13 mPa·s), indicating substantial granule disruption during the heating-holding phase. Conversely, AgNO_3_ treatment markedly reduced breakdown viscosity (771 ± 28 mPa·s), suggesting that the ionic environment either stabilized granule integrity or fundamentally altered the swelling mechanism [38,39,40]. Balet et al. (2019) noted that breakdown viscosity reflects the stability of starch paste under thermal and mechanical stress, with higher values indicating greater susceptibility to granule rupture [17]. Pure soybean flour (0T100S) exhibited minimal pasting activity in both solvent systems, with peak viscosities of 169 ± 15 mPa·s (AgNO_3_) and 257 ± 25 mPa·s (distilled water). The absence of detectable pasting temperature (0 °C) confirms that soybean’s low starch content precludes meaningful gelatinization [41], as its functional contribution derives primarily from protein rather than carbohydrate components.

The flour blends demonstrated systematic variations in pasting parameters proportional to their teff content. As soybean incorporation increased from 10% to 40%, progressive decreases were observed across all viscosity parameters in both solvent systems. For instance, final viscosity in distilled water decreased from 6611 ± 36 mPa·s (100T0S) to 284 ± 13 mPa·s (60T40S), reflecting the dilution of starch-contributing teff with protein-rich soybean. Pasting temperature showed corresponding increases with higher soybean content (68.9 °C for 100T0S versus values approaching 68–72 °C in blends), suggesting that protein matrices may delay water accessibility to starch granules.

The setback viscosity, indicative of retrogradation tendency and gel-forming capacity, followed similar trends. Pure teff exhibited setback values of 951 ± 18 mPa·s (AgNO_3_) and 4137 ± 33 mPa·s (distilled water), while increasing soybean proportions progressively reduced these values [42,43]. According to Balet et al. (2019), lower setback viscosities correlate with reduced amylose reassociation during cooling, which is potentially beneficial for products requiring extended shelf stability without excessive firming [17].

The substantial differences between solvent systems across all blend ratios show the crucial impact of ionic strength on the starch–protein matrix [44]. While AgNO_3_ provides insight into intrinsic starch properties by eliminating enzymatic interference, distilled water measurements better represent actual processing conditions.

### 2.4. Rheology

This study investigated the viscoelastic properties of teff, soybean, and their blends treated with AgNO_3_ and distilled water using oscillatory rheology (Table 4). Significant differences (*p* < 0.05) were observed across all treatments in storage modulus (G′), loss modulus (G″), tan δ, crossover points (G′ = G″), and their respective power law parameters (a, b, c).

The storage modulus (G′) values varied significantly (*p* < 0.05) among teff, soybean, and their blends depending on solvent type. Pure teff flour with distilled water recorded the highest G′ (1947.98 Pa), demonstrating strong elastic gel-like behaviour resulting from high starch content and efficient gelatinization in aqueous medium. In contrast, soybean flour exhibited substantially lower G′ values in both distilled water (132.71 Pa) and AgNO_3_ (48.96 Pa), confirming that protein-rich sources form weaker elastic networks than starch-rich materials.

Interestingly, the teff–soybean blends did not follow a simple linear trend with composition. When treated with AgNO_3_, the blends showed 90T10S (231.21 Pa), 80T20S (142.25 Pa), 70T30S (59.21 Pa), and 60T40S (45.84 Pa), representing a progressive decrease in gel strength with increasing soybean content. However, when treated with distilled water, the pattern was less predictable: 90T10S (103.83 Pa), 80T20S (67.12 Pa), 70T30S (57.66 Pa), and 60T40S (36.47 Pa). This suggests that while increasing soybean proportion generally weakens gel elasticity through starch dilution, the specific solvent–protein–starch interactions create complex rheological responses. These findings align with previous research [13,31,45] demonstrating that legume proteins hinder starch retrogradation and network strength.

The solvent effect was particularly pronounced for starch-rich formulations. Distilled water consistently produced higher G′ values than AgNO_3_ in pure teff and teff-dominant blends, confirming that water promotes starch swelling and network reinforcement. However, AgNO_3_ appears to interfere with starch–protein interactions, reducing gel elasticity [17,45]. This solvent-dependent behaviour indicates that ionic strength and polarity markedly influence the rheological behaviour of starch-based systems.

Similar patterns were observed for loss modulus (G″). Teff treated with water exhibited the highest G″ (142.85 Pa), while soybean-rich blends, particularly at 30% and 40% soybean inclusion, showed the lowest G″ values regardless of solvent. The tan δ values (G″/G′ ratio) were consistently less than unity across all flour samples, confirming that all formulations behaved as weak gels with elastic properties dominating over viscous properties [13]. Notably, teff blends with 30% and 40% soybean treated with AgNO_3_ displayed higher tan δ values than their water-treated counterparts, suggesting weaker gel networks with greater viscous contributions. This observation is consistent with [17], who reported that ion-mediated interactions reduce gel strength in starch–protein systems.

The power law parameters (a, b, c) characterize the frequency dependence of the viscoelastic moduli. Values of parameter ‘a’ (associated with G′) generally decreased with AgNO_3_ treatment compared to water, indicating reduced frequency sensitivity of the elastic component. Similarly, parameter ‘b’ (associated with G″) showed modifications with solvent type, reflecting altered viscous behaviour. The parameter ‘c’ (related to tan δ) exhibited notable changes, with soybean samples showing particularly pronounced increases under AgNO_3_ treatment, emphasizing how silver ions disrupt the protein–starch network structure.

The crossover modulus (where G′ = G″) provides insight into gel stability. Pure teff treated with distilled water showed the highest crossover point (1789.56 Pa), indicating a robust, stable gel structure. In contrast, most soybean-rich blends displayed substantially lower crossover values, particularly blends with 20% or more soybean in water (27.17–33.55 Pa), suggesting that soybean incorporation decreases structural integrity, especially in aqueous systems. This phenomenon has been reported previously [44,45] in starch–legume systems, where protein interference with starch retrogradation weakens gel formation.

For food industry applications, these findings have important implications. Teff-based products requiring strong gel strength (such as gluten-free baking or structured foods) benefit from water-based processing. However, for applications requiring softer textures or where protein enrichment is prioritized, soybean incorporation provides a viable strategy to modulate rheological properties. The distinct behaviour under AgNO_3_ treatment (considered as an ionic system) suggests that ionic modification (using food-grade ionic solvents) could serve as a processing tool for fine-tuning texture in specific applications, though this requires further investigation for food-grade implementation.

### 2.5. Texture Profile Analysis in Different Solvents

#### 2.5.1. Distilled Water

The texture properties (hardness, cohesiveness, springiness, chewiness, and resilience) of individual flours (teff and soybean) and their blends (Table 5) were significantly influenced by the incorporation of soybean. Highly significant differences (*p* < 0.0001) were observed among all sample types across all parameters, indicating substantial textural modifications resulting from blend composition.

Pure teff flour exhibited the highest hardness (7.60 N) and chewiness (4.87 N), reflecting its high starch content and dense gel structure. However, the incorporation of soybeans affected these properties. At 10% substitution level (90T10S), hardness and chewiness declined to 0.49 N and 0.57 N, representing approximately 94% and 88% reductions, respectively, compared to pure teff. This softening continued progressively: at 20% soybean substitution (80T20S), hardness decreased to 0.11 N, and at 30% substitution (70T30S), hardness reached 0.03 N, approaching the value of pure soybean (0.02 N). This progression demonstrates that the inclusion of soybeans rapidly dominates the texture profile, resulting in softer, less resilient structures. For food applications where pure teff’s natural hardness is undesirable, soybean incorporation offers an effective strategy to reduce hardness and chewiness, potentially improving consumer acceptability [46,47,48].

Cohesiveness exhibited an inverse relationship with hardness. Pure teff showed the lowest cohesiveness (0.46 N·s), while the highest value (3.58 N·s) was achieved at 30% soybean inclusion (70T30S). This pattern reflects fundamental differences in gel structure: teff’s starch-dominant matrix forms hard gels through strong retrogradation, resulting in rigid but less cohesive structures. When teff is blended with soybeans, the proteins interact with starch to form a modified gel matrix, while the soybean lipids prevent excessive retrogradation, resulting in softer, more elastic, and cohesive structures [43,49]. Intermediate cohesiveness values were observed in pure soybean (0.64 N·s) and the 10% soybean blend (0.83 N·s), indicating that moderate substitution levels optimize protein–starch interactions for cohesiveness.

Springiness, representing elastic recovery, was generally higher in teff and teff-based blends (1.21–2.13%) compared to pure soybeans (0.57%). The progressive increase in springiness with soybean addition (90T10S: 1.21%, 80T20S: 1.60%, 70T30S: 2.13%) suggests that the starch matrix from teff maintains structural integrity and elastic recovery even when blended with protein-rich soybean [43,46].

Resilience, measuring the ability to recover from deformation, showed an opposite pattern to hardness. Pure teff exhibited the lowest resilience (0.61), while pure soybean displayed the highest (2.22). Blends with higher soybean content demonstrated enhanced resilience, reflecting the ability of soybean protein to recover from compression and retain moisture during handling [46,50,51].

Overall, the incorporation of soybeans fundamentally modifies the textural characteristics of teff flour. The 10% soybean blend (90T10S) appears particularly promising, as it achieves substantial hardness reduction (94% decrease) while preserving moderate springiness (1.21%), offering a balanced texture profile. This formulation addresses the excessive hardness of pure teff while maintaining functional elastic properties. Higher soybean substitution levels (20–30%) produce progressively softer textures suitable for applications requiring minimal resistance, such as infant foods or soft bakery products. These findings confirm that teff–soybean blending is valuable for developing gluten-free, protein-enhanced food products that address consumer demand for functional, nutritious alternatives [17,44].

#### 2.5.2. Silver Nitrate (AgNO_3_)

Texture analysis of teff, soybean, and their blends using AgNO_3_ as solvent revealed significant differences (*p* < 0.05) across all parameters (Table 6). Pure teff exhibited the highest hardness (1.82 N) and chewiness (1.35 N·s), indicating a firm, compact gel structure even with AgNO_3_ treatment. Notably, pure soybean flour produced no measurable gel structure in AgNO_3_, likely because silver ions disrupt the limited protein–starch network formation capacity in the absence of sufficient starch, preventing gel consolidation.

Soybean incorporation progressively reduced hardness and chewiness in teff-based blends. At 10% soybean substitution (90T10S), hardness and chewiness decreased to 1.54 N and 1.07 N, respectively. At higher substitution levels, the softening effect became significant: 30% soybean (70T30S) yielded hardness of 0.19 N and chewiness of 0.09 N·s, while 40% soybean (60T40S) produced near-zero values (0.06 N hardness, 0.00 N·s chewiness). Cohesiveness values generally declined with increasing soybean content. Pure teff showed the highest cohesiveness (0.69 N), declining progressively to 0.36 N at 40% soybean inclusion. Springiness followed a similar trend, with pure teff and the 20% soybean blend showing the highest values (1.07% and 1.38%, respectively). In contrast, springiness was significantly reduced at 30% (0.64%) and 40% (0.18%) soybean inclusion. These results suggest that AgNO_3_ treatment reduces the elastic recovery potential of soybean-rich blends, likely due to silver ion interference with protein–starch gelation processes, which disrupts viscoelastic properties [44,52]. Zhang et al. [44] confirmed that ionic treatments can impair viscoelastic recovery in starch–protein systems.

Resilience values were highest in pure teff (1.18), decreasing substantially at higher soybean inclusion levels: 70T30S (0.44) and 60T40S (0.50).

These comparative analytical results provide important insights into the structural contributions of starch versus enzymatic activity in determining gel texture. The substantial difference between water and AgNO_3_ conditions for pure teff (7.60 N vs. 1.82 N hardness) demonstrates that endogenous enzyme activity significantly influences gel formation during thermal processing. For food applications, the water-based texture results are directly relevant, as they reflect conditions encountered during actual food processing. The data indicate that a 10–20% soybean substitution effectively reduces the excessive hardness of pure teff while preserving its functional elastic properties, making these blend ratios particularly suitable for applications such as gluten-free bakery products, infant foods, or soft snacks where a moderate texture is desirable [3,52,53,54,55].

### 2.6. Principal Component Analysis

**Figure 2:**
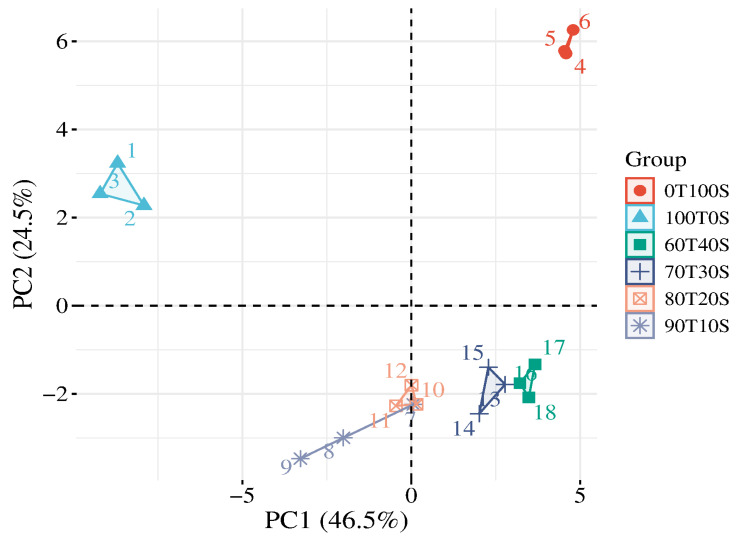
Factor map of the PCA performed on 18 samples and 45 variables. A total of 6 cluster groups were identified, corresponding to different samples. T—teff; S—soybean.

Principal component analysis of 45 functional, rheological, and textural parameters effectively differentiated teff and soybean flour blends (Figure 2). The first two principal components explained 71.0% of the total variance (PC1: 46.5%, PC2: 24.5%), providing clear separation based on compositional and processing-dependent properties.

PC1 represents differentiation based on whether functionality is driven by starch or protein. Pure teff flour, positioned at negative PC1 values, is associated with high viscosity development, strong gel formation, and firm texture—properties attributable to its starch-rich composition. Conversely, pure soybean flour at positive PC1 values is associated with an elevated water solubility index (WSI) and protein-related properties but exhibits minimal pasting capacity due to its limited starch content.

PC2 captures variation distinguishing pure ingredients from their blends. Both pure teff and pure soybean exhibit positive PC2 values, while all composite blends cluster at negative PC2 values. This pattern indicates non-additive functional interactions upon blending—the composite flours do not behave as simple mixtures of their parent components. This non-linearity likely results from competitive water binding, protein–starch complex formation, and interference with starch swelling by soybean proteins and lipids during hydration and thermal processing.

For food applications, products requiring high viscosity, strong gel networks, and firm texture should utilize teff-rich formulations (70–100% teff). Products emphasizing protein enrichment, dispersibility, or softer textures benefit from higher soybean inclusion (30–40%), though adequate viscosity may require hydrocolloid supplementation or careful optimization of processing conditions.

## 3. Materials and Methods

### 3.1. Gluten-Free Flour Preparation and Formulation

The experiment was conducted at the Department of Biotechnology and Food Analysis, Adaptive Food Systems Accelerator—Science Centre, Wroclaw, Poland. Teff (*Eragrostis tef*) was purchased from the local market in Gondar city, and soybeans were collected from the Gondar Agricultural Research Centre, Ethiopia. *Eragrostis tef* (Zuccagni) Trotter (Quyi—red teff) grains were manually cleaned to remove foreign matter and sun-dried to a constant weight. The dried grains were then milled using a local stone mill to produce whole-grain flour. Additionally, the soybeans (*Glycine max* (L.) Merr. var. Afgat) were manually cleaned, boiled in tap water, dehulled, and dried at 60 °C for 13 h to a constant weight. Then, they were milled into flour using a stone mill [18]. Finally, the flours were packed with a polyethene plastic bag, labelled, and kept at 4 °C until analysis to prevent oxidative degradation. Particle size of each sample flour and the blends were determined gravimetrically with sieves [56]. Then, 50 g of sample was sieved on a vibratory sieve shaker LPzE-2e (Multiserw Morek, Brzeźnica, Poland) at 0.65 mm vibration amplitude for 10 min, with screens of 80, 106, 125, 150, 180, and 355 microns. The percentage of each particle size fraction was reported only in three ranges, >355 μm, <250–355 μm, and <150 μm, as the weight of specific fractions was negligible. Each sample of flour was analyzed in triplicate.

### 3.2. Flour Blending

Soybean (S) flour was added to teff (T) flour in different ratios (0%, 10%, 20%, 30%, 40%), mixed at different ratios (for 10 min), and coded as 90T10S, 80T20S, 70T30S, and 60T40S. Flours were blended in 300 g batches with a planetary mixer (CLATRONIC KM 3400, CTC Clatronic, Warsaw, Poland) at the highest speed. Non-mixed flours were used as the control samples and were labelled as 100T0S and 0T100S, respectively. Each flour blend was packed in a Ziploc polyethene bag, labelled, and stored for further analysis.

### 3.3. Techno-Functional Properties of Blends

#### 3.3.1. Water Holding Capacity (WHC)

The water holding capacity (WHC) was determined according to the methods described in [15,30]. Approximately 0.5 g of dry matter from a sample of flour (WSWT) was mixed with 10 mL of distilled water. The dispersions were allowed to stand at room temperature for 24 h, and the supernatant was discarded. Then, the remaining sample was weighed (SWT). WHC (expressed as g of water held per g of sample dry matter) was calculated according to Equation (1). The analysis for each sample was carried out in triplicate.

WHC = SWT/WSWT(1)

#### 3.3.2. Water Absorption Capacity (WAC), Oil Absorption Capacity (OAC), Hydrophilic–Lipophilic Index (HLI)

Water absorption capacity (WAC) and oil absorption capacity (OAC) were determined by [15,30] with slight modifications. Briefly, 0.5 g of dried base (g DM) of the flour sample (SW) was taken and mixed with 10 mL of distilled water (g water) or corn oil (g oil) in pre-weighted (TW) centrifuge tubes and occasionally vortexing (Vortex 06-MXS, Stargard, Poland) for 30 s. The dispersions were allowed to stand at room temperature for 10 min before the process was repeated twice. They were then centrifuged at 3000 rpm (MPW-251) for 25 min. Then, the supernatant was discarded, and the sample tubes (STW) were oven drained (Vindon Scientific, Rochdale, UK) at 50 °C for 25 min and weighed. The difference between the initial volumes of distilled water/oil added to the sample and the volume obtained after filtration (WAC (g of water/g of DM), OAC (g of oil/g of DM), and HLI) were calculated as Equations (2) and (3). The analysis for each sample was carried out in triplicate.

WAC/OAC = (STW − TW)/SW(2)

HLI = WAC/OAC(3)

#### 3.3.3. Water Absorption Index (WAI), Water Solubility Index (WSI), Swelling Power SP

Water absorption index (WAI) and water solubility index (WSI), as well as swelling power SP of the flours, were measured as described in [30]. About 0.5 g of flour sample (W_S_) was dispersed in 10 mL of distilled water using tared centrifuge tubes, heated at 90 °C for 10 min in a water bath (MLL147, AJL Electronics, Krakow, Poland), cooled to room temperature, and centrifuged at 3000 rpm for 10 min. The supernatant was poured into a pre-weighed evaporating dish to determine its solid content, and the sediment was weighed (W_SS_). The weight of dry solids was recovered by evaporating the supernatant for 3 h. at 130 °C (W_DS_) and heated in an oven (SML, Zalmed, Łomianki, Poland). The analysis for each sample of flour was carried out in triplicate. WAI (g of water/g of DM), WSI (g of water/100 g of DM), and SP (g of water per g of DM) were calculated as Equations (4), (5), and (6), respectively.

WAI = Wss/Ws(4)

WSI = W_DS_/W_S_ (5)

SP = W_SS_/((W_S_ − W_DS_)) (6)

### 3.4. Pasting Properties: Viscoamylographic Tests (RVA)

The pasting characteristics of the sample flours and their blends were determined according to [57,58,59] using the Rapid Visco Analyser Starch Master2 (Newport Scientific, Sydney, Australia). A standard amount of sample and solvents was adjusted by ThermoCline v. 3.17.5.15 software. Distilled water and silver nitrate (0.1 M AgNO_3_) were used as solvents to examine the pasting characteristics of individual sample flours and blends. Silver nitrate was employed as an α-amylase inhibitor following established methodology [35,58], allowing characterization of intrinsic starch pasting behaviour without interference from endogenous enzymatic activity. The temperature profile implemented in all the blends follows the parameters: initial temperature at 50 °C for 2 min; heating from 50 °C to 95 °C in 4 min; then holding for 5 min; and finally cooling for 4 min to 50 °C. The peak viscosity (PV), trough viscosity (TV), breakdown viscosity (BV), final viscosity (FV), setback viscosity (SV), peak time (min), and pasting temperature (°C) were measured and calculated. The analysis for each sample was carried out in triplicate.

### 3.5. Rheological Analysis: Viscoelastic Behaviour: Oscillatory Tests

Rheological properties of sample flours were measured after the pasting property analysis. After the heating–cooling cycle, the paste was placed in 3 mL plastic containers and allowed to rest for 10 min before being transferred to the rheometer plate. A 40 mm parallel plate geometry with serrated steel surfaces was used, with a 1 mm working gap. Dynamic oscillatory tests of the blends were conducted at a temperature of 25 °C, controlled by a KNX2002 thermal controller. The tests were performed using an Anton Paar MC102 rheometer (Anton Paar, Graz, Austria). A frequency sweep ranging from 1 to 11 Hz was performed in the linear viscoelastic region at a constant stress of 1 Pa, illustrating viscoelastic behaviour through the storage or elastic modulus (G′) and the loss or viscous modulus (G″). The testing points were modified to conform to a power law, facilitating the estimation of coefficients a, b, and c. The analysis for paste samples was carried out twice in triplicate, in total six replicates.

### 3.6. Texture Gel Analysis

To generate the gels for texture analysis, the paste obtained from the Rapid Visco Analyser (RVA) was used to prepare the gels. The obtained paste was stored at 4 °C for 24 h after being moulded into cylindrical moulds with a 20 mm diameter. A texture profile analysis (TPA) test was performed on the gels using a texture analyser (FC200STAV500/300 AXIS, Gdansk, Poland) after the gels were demolded and set to a height of 20 mm. AXIS FM software version v.2_18 was used to collect the data. For every sample, the analysis was performed six times.

### 3.7. Statistical Analysis

All results are expressed as mean values accompanied by standard deviation (± SD). The variance of the results (ANOVA) and PCA analysis were evaluated using Stat-Graphics Centurion software (Centurion XVIII version, StatPoint Technologies, Inc., Warrenton, VA, USA). ANOVA was performed with a previous normality check of the data using a *p*-value < 0.05 significance level. Differences between group means were assessed for statistical significance at a confidence level of *p* ≤ 0.05 using Tukey’s Multiple Range Test. PCA bootstrap graphs were prepared using the SRPlot platform [59].

## 4. Conclusions

The systematic evaluation of functional, rheological, and textural properties across varying blend ratios demonstrates that these two naturally gluten-free ingredients exhibit remarkable synergy when properly combined. Teff’s starch functionality—manifested through exceptional water absorption index, swelling power, and robust gel formation—provides the structural foundation essential for gluten-free products. Complementarily, soybeans’ elevated protein content, enhanced water-holding capacity, and solubility address the nutritional deficiencies and processing limitations inherent in cereal-based formulations. The particle size–functionality relationship further demonstrates that optimization extends beyond simple compositional ratios and includes milling parameters, with coarse fractions enhancing binding properties and fine fractions promoting hydration.

Texture analysis confirms that a 10–20% soybean substitution achieves an optimal balance, reducing teff’s excessive hardness (a 94% reduction at 10% substitution) while preserving the functional elastic properties essential for product quality.

These findings establish teff–soybean composite flours as a promising platform for developing protein-enriched (>20% increase), nutritionally dense, gluten-free products with tailored textural characteristics. The blends are particularly suited for applications in bakery products, extruded snacks, infant foods, and therapeutic nutrition where both protein fortification and acceptable texture are critical. Future research should prioritize sensory optimization, consumer acceptance studies, shelf-life stability assessment, and large-scale production validation to facilitate commercial implementation of these functionally remarkable composite flours.

Future research should incorporate complementary analytical techniques including differential scanning calorimetry (DSC) for thermal transition characterization and scanning electron microscopy (SEM) for granule morphology visualization to further elucidate the structural basis of the functional properties reported herein. Additionally, validation of nutritional enhancement through compositional analysis and bioavailability studies would strengthen the application potential of these composite flours.

## Figures and Tables

**Table 1:** Absorption parameters of teff and soybean flours and their blends.

Samples	WHC	WAC	WAI	SP	HLI	WSI	OAC
	g H_2_O/g DM					g H_2_O/100 g DM	g H_2_O/g DM
0T100S	5.54 ± 0.55 ^b^	2.63 ± 0.20 ^b^	3.15 ± 0.32 ^a^	5.27 ± 0.27 ^a^	1.39 ± 0.14 ^a^	40.18 ± 3.73 ^e^	1.89 ± 0.05 ^d^
100T0S	4.60 ± 0.23 ^a^	2.85 ± 0.19 ^b^	5.62 ± 0.38 ^c^	6.18 ± 0.40 ^b^	1.56 ± 0.21 ^a^	9.05 ± 0.63 ^a^	1.84 ± 0.14 ^d^
90T10S	4.72 ± 0.16 ^a^	0.69 ± 0.08 ^a^	4.73 ± 0.16 ^b^	5.26 ± 0.18 ^a^	32.07 ± 5.98 ^c^	10.01 ± 0.40 ^ba^	0.02 ± 0.00 ^a^
80T20S	4.93 ± 0.44 ^ab^	0.77 ± 0.02 ^a^	4.54 ± 0.11 ^b^	5.26 ± 0.15 ^a^	2.80 ± 0.41 ^a^	13.68 ± 1.16 ^cb^	0.28 ± 0.04 ^c^
70T30S	4.69 ± 0.28 ^a^	0.59 ± 0.01 ^a^	4.56 ± 0.11 ^b^	5.39 ± 0.16 ^a^	2.98 ± 0.29 ^a^	15.47 ± 0.83 ^cd^	0.20 ± 0.02 ^bc^
60T40S	4.73 ± 0.66 ^a^	0.72 ± 0.04 ^a^	4.50 ± 0.06 ^b^	5.61 ± 0.06 ^ab^	21.42 ± 2.16 ^b^	19.93 ± 0.23 ^d^	0.04 ± 0.01 ^ab^

Lower case letters mean significantly different in column (*p* = 0.05); T—teff; S—soybean; WHC—water holding capacity; WAC—water absorption capacity; OAC—oil absorption capacity; HLI—hydrophilic–lipophilic index; WAI—water absorption index; WSI—water soluble index; SP—swelling power, DM—dry matter.

**Table 2:** Pearson correlation between particle size distribution and functional properties of soybean, teff, and blend flours.

100	100T0S		90T10S		80T20S		70T30S		60T40S	0T100S
% >355 μm	58.67 ± 3.53 ^a^		52.49 ± 5.65 ^a^		74.82 ± 2.78 ^b^		80.43 ± 1.33 ^b^		88.26 ± 2.21 ^c^	94.81 ± 5.19 ^c^
% <355 μm–250 μm>	35.42 ± 3.51 ^e^		39.43 ± 3.77 ^e^		21.15 ± 3.19 ^d^		16.32 ± 1.01 ^c^		9.49 ± 2.12 ^b^	2.12 ± 1.34 ^a^
% <150 μm	3.71 ± 0.43 ^bc^		8.54 ± 2.19 ^d^		4.34 ± 0.47 ^c^		3.54 ± 0.42 ^bc^		2.57 ± 0.21 ^b^	0.67 ± 0.64 ^a^
	**WHC**	**WAC**	**OAC**	**HLI**	**WAI**	**WSI**	**SP**	**>0.355 μm**	**0.255 μm** **–0.355 μm**	**<0.25 μm**
<0.2 μm	−0.57	−0.47	−0.53	0.67	0.52	−0.74	−0.14	−0.88 *	0.84 *	1.00
<0.25 μm–0.355 μm>	−0.64	0.00	−0.08	0.34	0.78	−0.81 *	0.35	−1.00 *	1.00	
>0.355 μm	0.64	0.07	0.15	−0.40	−0.76	0.82 *	−0.28	1.00		
SP	−0.50	0.54	0.46	−0.22	0.70	−0.37	1.00			
WSI	0.92 *	0.38	0.45	−0.29	−0.92 *	1.00				
WAI	−0.91 *	−0.06	−0.15	0.12	1.00					
HLI	−0.33	−0.52	−0.61	1.00						
OAC	0.48	0.99 *	1.00							
WAC	0.40	1.00								
WHC	1.00									

Lower case letters mean significantly different in row (*p* = 0.05); T—teff; S—soybean; WHC—water holding capacity; WAC—water absorption capacity; OAC—oil absorption capacity; HLI—hydrophilic–lipophilic index; WAI—water absorption index; WSI—water soluble index; SP—swelling power. *—marks correlation value over 0.8.

**Table 3:** Pasting characteristics of flours and their blends using distilled water and AgNo_3_ as a solvent.

Samples	Solvent	PV mPa·s	TV mPa·s	BV mPa·s	FV mPa·s	SBV mPa·s	PT s	Ptemp °C
0T100S	AgNo_3_	169 ± 15 ^d^	109 ± 13 ^d^	60 ± 90 ^d^	234 ± 21 ^d^	125 ± 10 ^d^	7.0 ± 0 ^a^	0 ± 0 ^c^
	D. Water	257 ± 25 ^c^	116 ± 90 ^c^	142 ± 16 ^c^	611 ± 41 ^c^	496 ± 32 ^c^	7.0 ± 0 ^a^	0 ± 0 ^c^
100T0S	AgNo_3_	1839 ± 30 ^b^	1068 ± 4 ^b^	771 ± 28 ^b^	2019 ± 14 ^b^	951 ± 18 ^b^	5.4 ± 0.10 ^b^	76.2 ± 0.5 ^a^
	D. Water	12,198 ± 133 ^a^	2473 ± 23 ^a^	9725 ± 13 ^a^	6611 ± 36 ^a^	4137 ± 33 ^a^	7.5 ± 0.31 ^a^	68.9 ± 0.3 ^b^
90T10S	AgNo_3_	1714 ± 505 ^b^	994 ± 19 ^b^	720 ± 28 ^b^	1817 ± 35 ^b^	822 ± 15 ^b^	5.48 ± 0.18 ^b^	75.85 ± 0.35 ^a^
	D. Water	644 ± 60 ^c^	455 ± 40 ^c^	190 ± 30 ^c^	1037 ± 11 ^c^	583 ± 70 ^c^	5.31 ± 0.03 ^b^	71.00 ± 0 ^b^
80T20S	AgNo_3_	1248 ± 63 ^b^	805 ± 33 ^b^	443 ± 28 ^b^	1403 ± 64 ^b^	598 ± 32 ^b^	5.31 ± 0.03 ^b^	76.63 ± 0.05 ^a^
	D. Water	331 ± 80 ^c^	263 ± 50 c	68 ± 30 ^c^	616 ± 14 ^c^	353 ± 80 ^c^	5.00 ± 0.00 ^b^	70.33 ± 0.58 ^b^
70T30S	AgNo_3_	638 ± 70 ^d^	476 ± 20 ^d^	163 ± 70 ^d^	797 ± 60 ^d^	321 ± 60 ^d^	5.22 ± 0.04 ^b^	77.98 ± 0.48 ^a^
	D. Water	218 ± 20 ^e^	190 ± 15 ^e^	28 ± 40 ^e^	447 ± 33 ^e^	256 ± 16 ^e^	4.73 ± 0.06 ^b^	68.33 ± 0.58 ^b^
60T40S	AgNo_3_	462 ± 46 ^e^	368 ± 43 ^e^	94 ± 40 ^e^	575 ± 43 ^e^	207 ± 30 ^e^	5.07 ± 0.07 ^b^	82.90 ± 5.5 ^a^
	D. Water	129 ± 90 ^f^	125 ± 70 ^f^	3 ± 30 ^f^	284 ± 13 ^f^	159 ± 90 ^f^	4.67 ± 0.12 ^b^	68.67 ± 1.15 ^b^

Lower case letters mean significantly different in column (*p* = 0.05); T—teff; S—soybean; PV—pasting viscosity; TV—trough viscosity; BV—breakdown viscosity; FV—final viscosity; SBV—setback viscosity; PT—pasting time; Ptemp—pasting temperature; D. Water—distilled water.

**Table 4:** Comparative viscoelasticity characteristics of teff, soybean, and their blends using solvent distilled water and AgNO_3_.

Sample	Treatment	G′ [Pa]	a	G″ [Pa]	B	Tan δ	c	G′ = G″ [Pa]
0T100S	AgNO_3_	48.96 ± 7.93 ^ab^	0.01 ± 0.00 ^a^	9.99 ±2.09 ^abc^	0.18 ±0.02 ^a^	0.20 ± 0.01 ^c^	0.18 ± 0.02 ^ef^	2.23 ± 1.00 ^a^
	D. Water	132.71 ± 33.66 ^abcd^	0.15 ± 0.01 ^cde^	32.54 ± 7.62 ^e^	0.24 ±0.02 ^b^	0.25 ± 0.01 ^d^	0.10 ± 0.01 ^a^	29.02 ± 6.47 ^a^
100T0S	AgNO_3_	195.27 ± 58.83 ^cd^	0.12 ± 0.02 ^c^	25.22 ±3.91 ^de^	0.26 ± 0.02 ^bc^	0.14 ± 0.02 ^b^	0.14 ± 0.01 ^bcde^	216.85 ± 48.75 ^c^
	D. Water	1947.98 ± 129.94 ^e^	0.07 ± 0.01 ^b^	142.85 ± 15.99 ^f^	0.18 ±0.01 ^a^	0.07 ± 0.01 ^a^	0.11 ± 0.01 ^ab^	1789.56 ± 158.40 ^d^
90T10S	AgNO_3_	231.21 ± 93.95 ^d^	0.11 ± 0.03 ^c^	29.86 ± 7.92 ^e^	0.24 ± 0.05 ^bc^	0.14 ± 0.03 ^b^	0.13 ± 0.02 ^abc^	212.08 ± 89.03 ^bc^
	D. Water	103.83 ± 12.59 ^abc^	0.14 ± 0.01 ^cde^	16.59 ± 2.37 ^bcd^	0.28 ± 0.01 ^cd^	0.16 ± 0.01 ^b^	0.14 ± 0.02 ^bcde^	62.85 ± 6.79 ^a^
80T20S	AgNO_3_	142.25 ± 19.67 ^bcd^	0.12 ± 0.01 ^cd^	21.25 ± 3.62 ^cde^	0.26 ± 0.01 ^bc^	0.15 ± 0.01 ^b^	0.14 ± 0.02 ^abcd^	110.10 ± 16.09 ^ab^
	D. Water	67.12 ± 10.94 ^ab^	0.13 ± 0.02 ^cd^	10.44 ± 1.44 ^abc^	0.30 ± 0.01 ^de^	0.16 ± 0.01 ^b^	0.17 ± 0.02 ^de^	33.55 ± 2.84 ^a^
70T30S	AgNO_3_	59.21 ± 6.19 ^ab^	0.16 ± 0.01 ^e^	12.12 ± 1.23 ^abc^	0.30 ± 0.01 ^de^	0.21 ± 0.01 ^c^	0.13 ± 0.01 ^abc^	33.67 ± 2.53 ^a^
	D. Water	57.66 ± 3.35 ^ab^	0.14 ± 0.02 ^cde^	9.20 ± 0.46 ^ab^	0.30 ± 0.01 ^de^	0.16 ± 0.01 ^b^	0.16 ± 0.02 ^cde^	27.17 ± 2.62 ^a^
60T40S	AgNO_3_	45.84 ± 6.17 ^ab^	0.15 ± 0.02 ^de^	8.94 ± 1.23 ^ab^	0.31 ± 0.01 ^de^	0.20 ± 0.01 ^c^	0.16 ± 0.03 ^cde^	21.51 ± 1.36 ^a^
	D. Water	36.47 ± 4.60 ^a^	0.12 ± 0.03 ^cd^	5.27 ± 0.80 ^a^	0.33 ± 0.01 ^e^	0.14 ± 0.01 ^b^	0.21 ± 0.03 ^f^	15.70 ± 1.73 ^a^

Lower case letters mean significantly different in column (*p* = 0.05); T—teff; S—soybean; D. Water—distilled water.

**Table 5:** Texture properties of teff–soybean composite flours using distilled water as solvent.

Sample	Hardness N	Cohesiveness	Springiness	Chewiness N	Resilience
0T100S	0.02 ± 0.00 ^a^	0.64 ± 0.15 ^b^	0.57 ± 0.56 ^ab^	0.01 ± 0.01 ^a^	2.22 ± 1.23 ^c^
100T0S	7.60 ± 0.78 ^d^	0.46 ± 0.11 ^a^	1.39 ± 0.61 ^c^	4.87 ± 2.84 ^d^	0.61 ± 0.04 ^a^
90T10S	0.49 ± 0.17 ^b^	0.83 ± 0.40 ^bc^	1.21 ± 0.67 ^c^	0.57 ± 0.61 ^b^	0.57 ± 0.07 ^a^
80T20S	0.11 ± 0.11 ^ab^	1.50 ± 1.47 ^cd^	1.60 ± 1.24 ^c^	0.47 ± 0.70 ^b^	0.60 ± 0.22 ^ab^
70T30S	0.03 ± 0.01 ^a^	3.58 ± 4.71 ^d^	2.13 ± 2.43 ^c^	0.53 ± 0.77 ^b^	1.69 ± 1.07 ^bc^
60T40S	NA	NA	NA	NA	NA

Lower case letters mean significantly different in column (*p* = 0.05); T—teff; S—soybean; NA—not available.

**Table 6:** Texture properties of teff–soybean composite flours using AgNO_3_ as solvent.

Sample	Hardness N	Cohesiveness	Springiness	Chewiness N	Resilience
0T100S	1.82 ± 0.19 ^d^	0.69 ± 0.02 ^d^	1.07 ± 0.08 ^b^	1.35 ± 0.16 ^d^	1.18 ± 0.72 ^c^
100T0S	NA	NA	NA	NA	NA
90T10S	1.54 ± 0.47 ^c^	0.65 ± 0.02 ^c^	1.09 ± 0.14 ^b^	1.07 ± 0.26 ^c^	0.94 ± 0.61 ^c^
80T20S	0.91 ± 0.08 ^b^	0.86 ± 0.28 ^d^	1.38 ± 0.36 ^c^	1.11 ± 0.61 ^c^	0.69 ± 0.09 ^b^
70T30S	0.19 ± 0.04 ^a^	0.56 ± 0.38 ^b^	0.64 ± 0.67 ^ab^	0.09 ± 0.10 ^b^	0.44 ± 0.09 ^a^
60T40S	0.06 ± 0.01 ^a^	0.36 ± 0.10 ^a^	0.18 ± 0.15 ^a^	0.00 ± 0.00 ^a^	0.50 ± 0.18 ^ab^

Lower case letters mean significantly different in column (*p* = 0.05); T—teff; S—soybean; NA—not available.

## Data Availability

The raw data supporting the conclusions of this article are available in the main text of the manuscript.
